# Supplementary figures and images for: Climate and the Parasite Paradox: Tick–Host Networks Depend on Gradients of Environmental Overlap
Source: Pathogens. 2025 Oct 10;14(10):1025. doi: 10.3390/pathogens14101025 (PMC12567382; doi:10.3390/pathogens14101025)

Jaccard index per parasite species and region

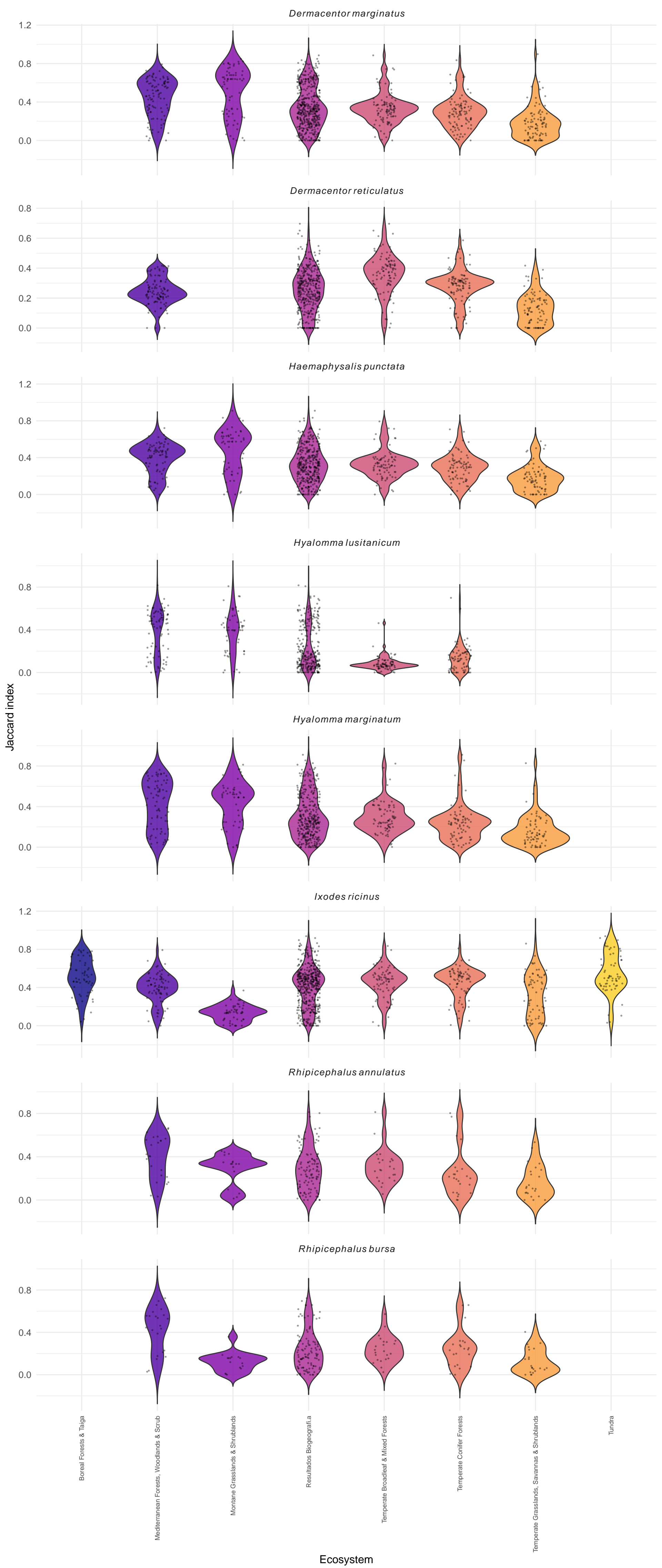

Supplement: Supplementary file 1 [file pathogens-14-01025-s001.zip › Supplementary Figure 1.pdf]
